# Association between the diagnosis of diet-related non-communicable diseases and the use of nutritional labeling among Mexican, Mexican American, and non-Mexican American adults: a cross-sectional study from the International Food Policy Study 2021–2022

**DOI:** 10.21203/rs.3.rs-7521567/v1

**Published:** 2025-09-23

**Authors:** Isabel García-Perfecto, Alejandra Contreras-Manzano, Kathia Larissa Quevedo, Christine M. White, Lana Vanderlee, Rachel E. Davis, Cecilia I. Oviedo-Solís, James F. Thrasher, Simón Barquera, David Hammond, Claudia Nieto, Dai Fang, Alejandra Jáuregui

**Affiliations:** National Institute of Public Health; National Institute of Public Health; National Institute of Public Health; University of Waterloo; Université Laval; University of South Carolina; National Institute of Public Health; University of South Carolina; National Institute of Public Health; University of Waterloo; National Institute of Public Health; University of South Carolina; National Institute of Public Health

**Keywords:** non-communicable diseases, nutrition label, front of pack labeling, warning labels, critical nutrients

## Abstract

**Background:**

Diet-related non-communicable diseases (NCDs) are a leading cause of morbidity and mortality worldwide. Front-of-package warning labels (WLs) and nutrition facts labels (NFLs) have been implemented to help consumers make healthier choices, yet little is known about their use among individuals with NCDs or across different population groups. Understanding these patterns is essential to evaluate labeling policies and their potential to support healthier diets. This study aimed to examine the association between NCD diagnosis and WL use among Mexican adults, and to compare NFL use across Mexican, Mexican American, and non-Mexican American adults.

**Methods:**

This study used cross-sectional data from the 2021 and 2022 International Food Policy Study. We analyzed self-reported WL and NFL use and NCD diagnoses (diabetes, hypertension, heart disease, high cholesterol, cancer) among adults in Mexico, Mexican Americans, and non-Mexican Americans. Multivariate logistic regression models assessed associations between label use and NCD status, adjusting for sociodemographic and health-related confounders.

**Results:**

Among 23,951 adults, NFL use was highest among non-Mexican Americans (80.1%) and lowest among Mexicans (69.8%, p < 0.01). NFL use was significantly associated with diabetes and multiple NCDs in non-Mexican Americans. In Mexico, WL use (77.4%) exceeded NFL use. Mexicans with diabetes and high cholesterol reported that “Excess Sugar” and “Excess Sodium” labels were particularly helpful.

**Conclusions:**

Labeling use varied across populations and NCD status. Findings highlight the importance of promoting interpretive front-of-package labels, especially among individuals with NCDs, to encourage healthier food choices and reduce diet-related disease burden.

## 1. Background

Non-communicable diseases (NCDs) are the leading cause of global mortality, responsible for approximately 15 million deaths annually among adults aged 30–60 years (1). Mexico and the United States have some of the highest adult obesity prevalences globally (32.2% and 41.6% respectively) (2), diabetes (12.4% and 14.7%) (3, 4), hypertension (29.9% and 47.3%) (5, 6) and other diet-relation conditions (7). The excessive consumption of foods high in critical nutrients (e.g., sugars, fats, sodium) has emerged as a major dietary driver of NCD risk (1).

Interpretive nutrition labels, such as front-of-package warning labels (WLs), aim to reduce unhealthy food consumption and support NCD management by providing simple, prominent information on excessive nutrients (8, 9). Nutrition facts labels (NFLs), mandatory in both Mexico and the U.S. (10, 11), offer detailed quantitative information but require greater nutritional literacy. U.S. data show NFL usage rates of 61.6% among non-Mexican Americans and 60.0% among Mexican Americans (12). The 2017 International Food Policy Study (IFPS) survey found that NFL use among US Latinos (57%) was lower than among non-Latino Whites (70%) but higher than among Mexicans (38%) (13).

Recent label policy changes include Mexico’s 2020 implementation of octagonal WLs and U.S. NFL updates in 2021 (10, 11). Some studies have found lower usage of the NFLs among Mexican Americans compared to non-Latino U.S. residents; a discrepancy that has been attributed to language barriers that hinder interpretation in these subpopulations (13, 14).

To address the challenges of interpreting and using the NFL, front-of-package labeling systems aim to clearly and concisely communicate nutrition information about packaged foods at the point of product selection and consumption. In 2020, Mexico introduced black octagonal warning labels on the front of the packages that clearly identify foods with “excessive” levels of calories, sugars, sodium, saturated fat, or trans-fat, as well as warnings for products containing non-nutritive sweeteners (10). Mexican consumers have shown a high level of understanding and acceptance of this labeling system (14).

Some subpopulations may be more prone to using food labels, particularly those who have diet-related illnesses that require closer monitoring of nutritional practices. Previous reports from the United States and Korea have shown that NFL use is associated with healthier food decision-making among individuals with NCDs (15, 16). These studies, primarily cross-sectional, suggest that individuals with NCDs are more likely to use NFLs than those without NCDs, suggesting a positive association between NFL use and food decision-making among people with NCDs.

In contrast, in Mexico, individuals with three or more NCDs were less likely to use the GDA label (OR: 0.34, p < 0.01) compared with 0, 1, 2 or 3 NCDs (17). This finding highlights a potential gap in the effectiveness of labeling systems for individuals with multiple NCDs. Different NCDs may require specific types of nutritional information; for example, people with diabetes may seek nutrition information to identify products high in sugar, while people with hypertension may be more concerned about sodium content of foods. Understanding these nuances can help tailor labeling policies to better meet the needs of groups with different diet-related NCDs.

This study aimed to assess the association between the diagnosis of diet-related NCDs and the use of WL among Mexican adults, as well as the perceived usefulness of each WL for making healthy food choices.

A secondary objective was to compare the association between having NCDs and using NFLs among three groups: non-Mexican American, Mexican American, and Mexican adults.

## 2. Methods

### 2.1 Study Design

Data was collected through the International Food Policy Study (IFPS), an online survey conducted annually among adults in five countries during November and December. Secondary data analyses were performed using 2021 and 2022 data from adults in the Mexico and United States arms of the IFPS.

### 2.2 Participants

Participants were recruited through the Nielsen Global Consumer Panel and Qualtrics online panels with their partners using non-probability sampling methods with age and sex quotas to obtain a diverse sample that better represented each country’s demographic proportions. Eligibility criteria included being 18 years or older and residing in one of the selected countries. Oversampling strategies were implemented differently by country: in Mexico, participants with the lowest education levels were oversampled to improve representation of this group, while in the United States, there was specific oversampling of Mexican American participants.

For this study, surveyed adults were categorized into three subpopulations: 1) “Mexicans” (adults residing in Mexico), 2) “Mexican Americans” (US adults of Mexican heritage), and 3) “non-Mexican Americans” (other US ethnic groups including White, Black/African American, Asian/Pacific Islander, Native American, and Hispanic/Latino individuals not of Mexican origin). Participants provided informed consent before the survey and received compensation according to their panel’s standard incentive structure (28). Complete IFPS study methods are detailed in the Technical Reports (18).

### 2.3 Measures

#### Use of nutrition labels

NFL use was assessed with the question, “How often do you use this type of food label when deciding to buy a food product?” accompanied by an image of the country-specific NFL. Response options were categorized as “not used” (never, rarely) and “used” (sometimes, often, all the time). Mexican participants were also asked a similar question regarding the use of WL, accompanied by an image of the ‘excess calories’ WL, with response options identical to those for the NFL. Use of WLs was only assessed in Mexico, and not in USA, because Mexico was the only country with a government endorsed front of package labeling scheme.

#### Perceived usefulness of warning labels

Additionally, in 2021, Mexican participants were shown images of all five WLs and asked to evaluate the usefulness of each WL with the question, “Which of these stamps, if any, has been most useful to choose healthier foods: excess calories, excess sugars, excess saturated fats, excess trans fats, excess sodium, none of the stamps have been useful, or all the stamps have been equally useful.

#### Type and number of diagnoses of diet-related non-communicable diseases (NCDs)

Participants reported diagnoses for six diet-related NCDs (“Has a doctor, nurse, or other health professional ever told you that you have or had…”), including the following: 1) hypertension or high blood pressure, 2) heart attack (myocardial infarction), 3) angina or coronary disease, 4) diabetes or high blood sugar, 5) high cholesterol, and 6) cancer (excluding skin cancer). Responses for each condition were categorized as “yes” or “no.”

The total number of self-reported NCDs was calculated by summing the number of conditions reported by each participant out of 6. This was then classified into three categories: “none,” “1 to 2” and “3 or more.”

#### Covariates

Covariates included sociodemographic characteristics and other relevant variables that may affect food choice, food intake, and NCD management. Sociodemographic characteristics included sex at birth, age group (18 to 30, 30, 30 to 39, 40 to 49, 50 to 59, 60 to 69, 70 to 100), and highest educational attainment using country-specific response options, recoded as: “low” (none, preschool, elementary school, middle school, high school, or basic normal education for Mexicans; none, 8th grade or lower, 9th grade, 10th grade, 11th grade, 12th grade or high school for Mexican Americans and non-Mexican Americans); “medium” (career and technical studies for Mexicans; associate degree for Mexican Americans and non-Mexican Americans); and “high” (bachelor’s degree or higher for both all respondents). Additionally, participants’ subjective income adequacy was assessed with the question, “Thinking about your total monthly income, how difficult or easy is it for you to make ends meet?” (Recoded as easy/very easy or neither easy nor difficult/difficult/very difficult. Body Mass Index (BMI) was calculated from self-reported weight and height, with participants classified into categories of BMI < 24.9 kg/m^2^, 25–29.9 kg/m^2^, ≥ 30 kg/m^2^, or missing. Nutrition knowledge was assessed using the question, “How would you rate your nutrition knowledge?” Responses were categorized as “none” (not at all knowledgeable), “low” (a little knowledgeable, somewhat knowledgeable), or “high” (very knowledgeable, extremely knowledgeable).

Several characteristics associated with the use of nutrition labels were also included as covariates and assessed through the following questions: Do you have children (under 18 years old) living in your household? (“yes” or “no”); This question was included because research has shown that the WL system is beneficial for parents when selecting healthy foods for their children (14). To assess the role in household food shopping, participants were asked, “How much of the food shopping do you do in your household?” Responses were recategorized as “primary shopper” (most) and “non primary shopper” (share equally with other(s); some, but less than other(s), none).

Participants who either declined to respond or answered ‘don’t know’ questions regarding the use of nutrition labeling (WL and NFL), diagnosis of NCDs, or adjustment covariates were excluded from the analytic sample (**Supplementary Fig. 1**).

### 2.4 Statistical Analysis

Descriptive statistics characterized the sample based on sociodemographic variables, dietary behaviors, and NCDs by analytic subpopulation of interest (i.e., Mexican, Mexican American, non-Mexican American). To estimate associations between diagnosis of specific NCDs and nutrition label use, adjusted logistic regression models were estimated separately for each subpopulation. These models independently estimated the associations between NFL or WL usage among Mexicans and each NCD diagnosis. Subsequently, a multivariate logistic regression model incorporating the entire analytic sample of non-Mexican Americans, Mexicans, and Mexican Americans was developed to evaluate the association between NFL usage and NCD diagnosis stratified by these subpopulations. Separate models were also developed based on the number of NCDs in each subpopulation. Furthermore, models treating the NCD variable as a continuous variable were run to assess how label usage and the perceived usefulness of warning labels varied with the increasing number of diseases. All models were adjusted for survey year, age, sex at birth, income adequacy, educational level, nutrition knowledge, presence of children under 18 years in the household, household shopping role, and BMI categories to control for potential confounding factors. Post-stratification weights were applied in all analyses. These were constructed using a raking algorithm with census population estimates for each country based on age group, sex, region, education, and ethnicity. Data analyses were performed using STATA version 16.0. Statistical significance was established with a p-value < 0.01 and a 99% confidence interval.

## 3. Results

The final analytic sample across 2021 and 2022 included 23,951 participants; of these 46.5% were non-Mexican Americans (n = 6,710), 25.5% were Mexican Americans (n = 6,101), and 28.0% were Mexicans (n = 11,140) (**Supplementary Fig. 1**). Table 1 displays the sociodemographic characteristics, nutrition labeling use, and prevalence of NCDs across analytic subpopulations.

In the adjusted independent logistic regression models for each NCD (Table 2) it was observed that non-Mexican Americans were 68% more likely to use the NFL when diagnosed with diabetes (AOR 1.68, 99% CI: 1.26–2.24) compared to those without an NCD diagnosis. Furthermore, non-Mexican Americans were 48% more likely to use the NFL when they had 3 or more NCD diagnoses (AOR 1.48, 99% CI: 1.07–2.06) compared to those without any NCD diagnosis. When analyzing the NCD variable as a continuous measure, it was found that for each diagnosed NCD among non-Mexican Americans, the likelihood of using the NFL increased by 16% (AOR 1.16, 99% CI: 1.02–1.32).

Table 3 presents the results of the association between NCD diagnosis and the perceived usefulness of WLs for selecting healthier foods among Mexican adults from the 2021 survey. Adults diagnosed with diabetes were more likely to perceive the “Excess Sugars” WL as the most useful when choosing healthier foods (AOR 1.65, 99% CI: 1.07–2.54) compared to those without any NCD diagnosis. Adults diagnosed with high cholesterol were more likely to perceive the “Excess Sodium” WL as the most useful for choosing healthier foods (AOR 1.89, 99% CI: 1.15–3.12) compared to those without any NCD diagnosis.

Regarding the number of diagnosed NCDs among Mexican adults (Table 3), those with 1 or 2 diagnoses were more likely to perceive the “Excess Sodium” WL as the most useful for choosing healthier foods (AOR 1.72, 99% CI: 1.08–2.73) compared to those without any NCD diagnoses. Furthermore, adults with 3 or more NCDs were more likely to perceive the “Excess Sugars” WL as the most useful for selecting healthier options (AOR 1.96, 99% CI: 1.04–3.71) compared to individuals without any diagnoses. In the regression model examining the number of NCDs as a continuous variable, it was observed that for each diagnosed NCD among Mexican adults, the likelihood of perceiving the “Excess Sodium” WL (AOR 1.21, 99% CI: 1.02–1.44) and the “Excess Sugars” WL (AOR 1.21, 99% CI: 1.05–1.39) as the most useful when choosing healthier foods increased.

## 4. Discussion

This study explored the use of the NFL and WLs across Mexicans, Mexican Americans, and non-Mexican Americans, with a focus on differences by NCD diagnosis. While a general trend of increased NFL use with more NCDs was observed, statistically significant differences were found only among non-Mexican Americans, particularly those with diabetes or three or more NCDs. This supports previous evidence suggesting that individuals with chronic conditions have greater nutrition awareness due to more frequent healthcare interactions, including dietary counseling (15, 19–21).

One study from the ENSANUT 2016 found that Mexican adults with hypertension, diabetes, or three or more NCDs tended to use the GDA front-of-package labeling system less than those without diagnoses (17). Additionally, ENSANUT 2018 data showed that individuals with NCDs were more likely to read the NFL than the GDA (22). This trend may be explained by the complexity of the GDA labeling system, which required nutritional knowledge, time, and mathematical calculations for proper use, making it difficult for most consumers to understand (22–25).

In contrast, our findings showed that WL use (77.5%) exceeded NFL use (69.8%) among Mexicans, and no significant differences were found in WL use by NCD status. This suggests that WLs are widely adopted regardless of diagnosis (14) regardless of NCD status. Supporting this, a prior Mexican study found no significant variation in WL use among those with diabetes or hypertension (26).

Previous studies in adult populations have reported that WLs encourage the selection of foods with lower amounts of critical nutrients (9). While our findings did not support the hypothesis that individuals with NCDs generally use WLs more frequently due to a heightened interest in avoiding excessive levels of critical nutrients to better manage their conditions (8), we observed a notableassociation when analyzing the perceived usefulness of WLs related to the critical nutrient to the diagnosed NCD.

For instance, individuals with diabetes were more likely to consider the “Excess sugars” label to be most useful when choosing healthy foods, and those with high cholesterol were more likely to find the “Excess sodium” label as most useful. Additionally, as the number of diagnosed NCDs increased, so did the likelihood of considering these two labels as the most useful for making informed food choices. This suggests a heightened awareness among individuals with NCDs regarding the importance of certain critical nutrients in the onset and progression of these diseases.

Similarly, our study found that among the Mexican population, the likelihood of considering the “Excess sodium,” “Excess trans-fat,” and “Excess sugars” WLs as useful doubled with an increasing number of NCDs. These findings align with evidence that warning label-type systems provide interpretative elements that quickly convey the nutritional quality of products, helping consumers choose those considered more beneficial for their health (27). Experimental studies conducted in various countries have shown that WLs enhance consumers’ ability to understand nutrition information and discourage the selection of products high in nutrients linked to NCDs (28–30).

Consistent with our findings, previous research based on 2017 IFPS data has shown that the overall U.S. population reports higher use of NFLs (70.00% vs 80.13% in our study) compared to the Mexican population (38.00% vs 69.81% in our study) and the U.S. Latino population (57.00% vs 77.61% in our study) (13). The greater use of NFLs among Americans could be attributed to the fact that NFLs is the primary tool available for obtaining nutrition information on packaged products, as there is no mandatory front-of-package labeling system in the United States (31). Conversely, Mexico’s recently implemented WL system might partly explain the lower use of NFLs in this population compared to subpopulations residing in the United States. However, an increase in NFL use has been observed in Mexico in recent years compared to the pre-implementation stage of the WL system (32). Despite these trends, further data are needed to determine why NFL use remains higher in the United States compared to Mexico.

One of the study’s main strengths is the large sample size, including NFL use data collected over two years, which allowed for rigorous statistical analyses. However, certain limitations should be acknowledged. Firstly, although oversampling of low education populations in Mexico and weighting techniques was applied to enhance representativeness, participant recruitment relied on non-probabilistic sampling, limiting generalizability to the national level.

Additionally, the cross-sectional design of our study prevents causal inferences between nutrition labels use and NCD presence. Furthermore, data on years since diagnosis and adherence to medical or nutritional treatment were not collected. These factors should be considered when interpreting our results, as they may influence both nutritional label use and NCD management.

### Conclusions

In conclusion, our findings highlight the potential of food labeling as an essential tool for individuals with health conditions. This underscores the need to promote the use of interpretive labels, such as the WLs, among populations with NCDs to support informed food choices that can contribute to both prevention and effective management of these conditions.

## Supplementary Files

This is a list of supplementary files associated with this preprint. Click to download.

- Supplementarymaterial.docx

## Figures and Tables

**Table 1:** Sociodemographic characteristics, nutrition labeling use, and diagnosis of NCDs by subpopulation. International Food Policy Study 2021 and 2022 (n = 23,951)

	Mexicans (Mx) (n = 11,140)	Mexican Americans (Ma) (n = 6,101)	Non-Mexican American (n-Ma) (n = 6,710)	*p*-value Mx vs Ma	*p*-value Mx vs n-Ma	*p*-value Ma vs n-Ma
	% (99% CI)	% (99% CI)	% (99% CI)			
**Sociodemographic characteristics**						
**Year**						
2021	48.56 (46.81–50.33)	49.74 (47.85–51.64)	50.99 (49.19–52.79)	0.240	0.013	0.220
2022	51.44 (49.67–53.19)	50.26 (48.36–52.15)	49.01 (41.21–50.81)			
**Sex at birth**						
Male	47.24 (45.49–48.99)	47.18 (45.29–49.08)	48.81 (47.01–50.61)	0.955	0.107	0.109
Female	52.76 (51.01–54.51)	52.82 (50.92–54.71)	51.19 (49.39–52.99)			
**Age group (years)**						
18 to 30	27.40 (25.97–28.88)	29.16 (27.50–30.88)	15.53 (14.21–16.94)	<0.01	<0.01	<0.01
30 to 39	23.68 (22.27–25.15)	27.70 (26.02–29.44)	14.79 (13.55–16.12)			
40 to 49	17.90 (16.72–19.14)	20.32 (18.82–21.89)	15.72 (14.46–17.06)			
50 to 59	18.94 (17.45–20.53)	13.48 (12.24–14.81)	17.85 (16.52–19.25)			
60 to 69	9.47 (8.28–10.80)	7.04 (6.07–8.16)	21.82 (20.37–23.35)			
70 to 100	2.61 (1.97–3.44)	2.30 (1.78–2.97)	14.30 (13.12–15.57)			
**Educational level**						
Low	74.88 (73.53–76.19)	65.29 (63.63–66.91)	50.84 (49.04–53.63)	<0.01	<0.01	<0.01
Medium	9.70 (8.66–10.86)	10.50 (9.74–11.30)	10.14 (9.40–10.93)			
			
High	15.41 (14.58–16.28)	24.22 (22.77–25.72)	39.02 (37.34–40.74)			
**Perceived income adequacy**						
Easy or very easy	10.38 (9.45–11.39)	25.84 (24.25–27.49)	42.45 (40.70–44.22)	<0.01	<0.01	<0.01
Neither easy nor difficult	40.22 (38.51–41.95)	38.32 (36.50–40.18)	30.35 (28.69–32.07)			
Difficult or very difficult	49.40 (47.64–51.17)	35.84 (34.02–37.71)	27.20 (25.60–28.85)			
**Presence of children (under 18) in the household**	50.72 (48.96–52.48)	45.26 (43.38–47.15)	25.65 (24.14–27.23)	<0.01	<0.01	<0.01
**Primary shopping role for household**	68.62 (66.98–70.22)	67.25 (65.45–69.01)	70.58 (68.88–72.22)	0.143	0.031	<0.01
**Nutrition knowledge**						
Not at all/ a little knowledgeable	44.21 (42.47–45.98)	33.56 (31.78–35.39)	34.41 (32.70–36.15)	<0.01	<0.01	0.026
Somewhat knowledgeable	46.19 (44.44–47.95)	42.37 (40.51–44.26)	39.81 (38.06–41.59)			
Very/extremely knowledgeable	9.60 (8.65–10.63)	24.07 (22.51–25.69)	25.78 (24.25–27.37)			
**BMI (kg/m^2^)**						
<24.9	32.31 (30.72–33.95)	30.81 (29.11–32.57)	37.49 (35.76–39.26)	<0.01	<0.01	<0.01
25.0 to 29.9	29.43 (27.84–31.07)	28.53 (26.85–30.27)	28.49 (26.91–30.12)			
>30	16.73 (15.42–18.12)	28.68 (26.99–30.43)	24.26 (22.74–25.84)			
Missing	21.53 (20.11–23.03)	11.98 (10.76–13.31)	9.76 (8.72–10.91)			
**Nutrition labeling use**						
**Nutrition facts table (NFL) use**				<0.01	<0.01	0.003
Used	69.81 (68.16–71.41)	77.61 (75.99–79.16)	80.13 (78.62–81.56)			
Not used	30.19 (28.59–31.84)	22.39 (20.84–24.01)	19.87 (18.44–21.38)			
**Front-of-pack warning label (WL) use**						
Used	77.46 (75.96–78.88)	-	-	-	-	-
Not used	22.54 (21.12–24.04)	-	-			
**Non-Communicable Diseases (NCDs)**						
**NCD diagnosis**						
Hypertension	23.50 (21.96–25.11)	25.22 (23.60–26.91)	37.33 (35.60–39.09)	0.051	<0.01	<0.01
Heart attack	2.62 (2.10–3.27)	3.08 (2.49–3.81)	5.16 (4.42–6.01)	0.172	<0.01	<0.01
Angina	2.66 (2.14–3.29)	3.60 (2.90–4.46)	5.36 (4.62–6.20)	0.010	<0.01	<0.01
Diabetes	13.00 (11.74–14.37)	15.17 (13.83–16.60)	16.10 (14.84–17.45)	0.003	<0.01	0.207
High cholesterol	24.34 (22.78–25.97)	21.16 (19.67–22.74)	32.45 (30.80–34.15)	<0.01	<0.01	<0.01
Cancer (excluding skin cancer)	2.03 (1.59–2.59)	4.20 (3.49–5.05)	7.12 (6.25–8.09)	<0.01	<0.01	<0.01
**Number of NCD diagnoses (categorical)**						
None	58.77 (56.99–60.53)	60.26 (58.38–62.10)	46.96 (45.17–48.77)	<0.01	<0.01	<0.01
1 or 2	35.16 (33.45–36.91)	30.69 (28.97–32.46)	39.78 (38.04–41.55)			
3 or more	6.07 (5.20–7.07)	9.06 (7.99–10.25)	13.25 (12.08–14.52)			

The p-value was calculated using Pearson’s chi-squared test.

**Table 2:** Association between the diagnosis of NCDs and the use of nutritional labeling in Mexican, Mexican American and non-Mexican American adults. IFPS 2021 and 2022

	Use of NFL			Use of WL
	Mexican	Mexican American	Non-Mexican American	Mexican
*n*	11,140	6,101	6,710	11,140
	AOR (99% CI)	AOR (99% CI)	AOR (99% CI)	AOR (99% CI)
**Diagnosis of NCDs** ^a^				
None	*ref*	*ref*	*ref*	*ref*
Hypertension	1.01 (0.82–1.24)	1.09 (0.86–1.38)	1.09 (0.88–1.35)	1.02 (0.82–1.27)
Heart attack	1.18 (0.66–2.08)	1.09 (0.61–1.97)	1.57 (0.96–2.57)	1.11 (0.61–2.02)
Angina	1.05 (0.63–1.76)	1.85 (0.94–3.63)	1.57 (0.97–2.53)	1.33 (0.73–2.43)
Diabetes	1.02 (0.78–1.34)	1.17 (0.87–1.56)	**1.68 (1.26–2.24)**	1.13 (0.84–1.52)
High cholesterol	0.94 (0.76–1.15)	1.21 (0.95–1.56)	1.06 (0.85–1.31)	0.90 (0.72–1.11)
Cancer	1.40 (0.77–2.56)	1.19 (0.71–1.99)	1.14 (0.78–1.69)	1.37 (0.74–2.57)
**Number of NCD diagnoses (categorical)** ^b^				
None	*ref*	*ref*	*ref*	*ref*
1 to 2	0.92 (0.77–1.11)	1.08 (0.86–1.35)	1.14 (0.91–1.42)	1.00 (0.82–1.22)
3 or more	1.07 (0.72–1.59)	1.37 (0.93–2.02)	**1.48 (1.07–2.06)**	1.06 (0.69–1.63)
**Number of NCD diagnoses (continuous)** ^c^	1.00 (0.87–1.14)	1.05 (0.91–1.22)	**1.16 (1.02–1.32)**	1.01 (0.92–1.12)

a Independent logistic regression models for each type of NCD and each subpopulation, adjusted for: survey year, sex, age group, educational level, income adequacy, children under 18 in the household, shopping role for household, nutrition knowledge, and BMI categories.

b Independent logistic regression models for each subpopulation, adjusted for: survey year, sex, age group, educational level, income adequacy, children under 18 in the household, shopping role for household, nutrition knowledge, and BMI categories.

c A logistic regression model was conducted using the NCD variable as a continuous measure.

Bold numbers indicate statistical significance (p < 0.01).

IFPS = International Food Policy Study (waves 2021 and 2022); NCDs = Non-Communicable Diseases; NFL = Nutrition Facts Label; WL = Warning Label; AOR = Adjusted Odds Ratio; CI = Confidence Interval.

**Table 3:** Association between the type and number of diagnosed NCDs and the perceived usefulness of warning labels for selecting healthier foods among Mexican adults. IFPS 2021 (n = 5,430)

	Excess calories	Excess sodium	Excess trans fats	Excess sugars	Excess saturated fats	None of the stamps have been useful	All of the stamps have been equally useful
	AOR (99% CI)	AOR (99% CI)	AOR (99% CI)	AOR (99% CI)	AOR (99% CI)	AOR (99% CI)	AOR (99% CI)
**Diagnosis of NCDs** ^a^							
None	*ref*	*ref*	*ref*	*ref*	*ref*	*ref*	*ref*
Hypertension	0.85 (0.53–1.38)	1.59 (0.97–2.61)	1.14 (0.65–2.03)	1.39 (0.99–1.95)	0.88 (0.58–1.32)	0.90 (0.62–1.31)	0.81 (0.60–1.08)
Heart attack	0.86 (0.35–2.15)	0.63 (0.19–2.06)	1.11 (0.71–1.74)	1.22 (0.49–3.05)	0.62 (0.21–1.87)	1.38 (0.62–3.10)	1.00 (0.48–2.07)
Angina	0.58 (0.20–1.67)	0.31 (0.09–1.04)	0.82 (0.23–2.92)	1.51 (0.67–3.39)	1.20 (0.49–2.91)	1.00 (0.33–3.01)	1.04 (0.48–2.25)
Diabetes	0.81 (0.41–1.62)	1.44 (0.75–2.77)	0.55 (0.23–1.29)	**1.65 (1.07–2.54)**	0.53 (0.27–1.04)	0.97 (0.59–1.60)	1.01 (0.69–1.47)
High cholesterol	0.64 (0.39–1.06)	**1.89 (1.15–3.12)**	1.17 (0.68–2.01)	1.34 (0.96–1.87)	0.79 (0.51–1.22)	0.80 (0.54–1.17)	0.98 (0.74–1.30)
Cancer	1.10 (0.26–4.66)	0.32 (0.07–1.40)	1.15 (0.37–3.56)	1.16 (0.45–3.00)	0.57 (0.15–2.10)	1.45 (0.48–4.40)	1.01 (0.43–2.38)
**Number of NCD diagnoses (categorical)** ^b^							
None	*ref*	*ref*	*ref*	*ref*	*ref*	*ref*	*ref*
1 or 2	0.72 (0.48–1.10)	**1.72 (1.08–2.73)**	1.50 (0.93–2.43)	1.25 (0.93–1.67)	0.83 (0.57–1.20)	0.93 (0.66–1.30)	0.87 (0.67–1.11)
3 or more	0.88 (0.34–2.31)	1.59 (0.64–3.39)	0.70 (0.24–2.06)	**1.96 (1.04–3.71)**	0.65 (0.26–1.62)	0.80 (0.37–1.74)	0.82 (0.46–1.46)
**Number of NCD diagnoses (continuous)** ^c^	0.85 (0.66–1.09)	**1.21 (1.02–1.44)**	1.00 (0.82–1.21)	**1.21 (1.05–1.39)**	0.85 (0.69–1.04)	0.95 (0.80–1.14)	0.96 (0.84–1.09)

a Independent logistic regression models for each type of NCD and each warning label, adjusted for: survey year, sex, age group, educational level, income adequacy, children under 18 in the household, shopping role for household, nutrition knowledge, and BMI categories.

b Independent logistic regression model for each warning label, adjusted for: survey year, sex, age group, educational level, income adequacy, children under 18 in the household, shopping role for household, nutrition knowledge, and BMI categories.

c A logistic regression model was conducted using the NCD variable as a continuous measure.

IFPS = International Food Policy Study (waves 2021 and 2022); NCDs = Non-Communicable Diseases, AOR = Adjusted Odds Ratio, CI = Confidence Interval

Bold numbers indicate statistical significance at a p-value < 0.01.

## Data Availability

The datasets analyzed during the current study are not publicly available. Data are available from the International Food Policy Study (contact: Dr. Christine White, University of Waterloo) upon reasonable request and with permission from the study team.

## Data Availability

The datasets analyzed during the current study are not publicly available. Data are available from the International Food Policy Study (contact: Dr. Christine White, University of Waterloo) upon reasonable request and with permission from the study team.

## Abbreviations

AOR: Adjusted odds ratio
BMI: Body mass index
CI: Confidence interval
ENSANUT: Encuesta Nacional de Salud y Nutrición
GDA: Guideline daily amounts
IFPS: International Food Policy Study
NFL: Nutrition facts label
NCD: Non-communicable disease
NCDs: Non-communicable diseases
NIH: National Institutes of Health
OR: Odds ratio
U.S.: United States
WL: Warning label
WLs: Warning labels
